# A Single Step Preparation of Photothermally Active Polyvinylidene Fluoride Membranes Using Triethyl Phosphate as a Green Solvent for Distillation Applications

**DOI:** 10.3390/membranes11110896

**Published:** 2021-11-19

**Authors:** Marcello Pagliero, Antonio Comite, Camilla Costa, Ilaria Rizzardi, Omar Soda

**Affiliations:** Membrane & Membrane Research Group, Department of Chemistry and Industrial Chemistry, University of Genoa, Via Dodecaneso 31, 16146 Genova, Italy; antonio.comite@unige.it (A.C.); camilla.costa@unige.it (C.C.); ilaria.rizzardi@edu.unige.it (I.R.); omar.soda@unige.it (O.S.)

**Keywords:** membrane distillation, carbon black, photothermal, green solvent, PVDF

## Abstract

Membrane distillation is a growing technology that can address the growing problem of water shortage. The implementation of renewable energy and a reduction in the environmental impact of membrane production could improve the sustainability of this process. With this perspective, porous hydrophobic polyvinylidene fluoride (PVDF) membranes were prepared using triethyl phosphate (TEP) as a green solvent, using the non-solvent induced phase separation technique. Different amounts of carbon black were added to dope solutions to improve the photothermal properties of the membranes and to enable direct heating by solar energy. By optimizing the preparation conditions, membranes with porosity values as high as 87% were manufactured. Vacuum membrane distillation tests carried out using a concentrated NaCl solution at 50 °C showed distillate fluxes of up to 36 L/m^2^ h and a complete salt rejection. Some preliminary studies on the photothermal performance were also conducted and highlighted the possibility of using such membranes in a direct solar membrane distillation configuration.

## 1. Introduction

The increasing demand for potable water in the last decades has forced academic and applied researchers to develop new technologies aimed at water purification. Several desalination techniques that exploit the largely available seawater have been improved or newly implemented.

In this context, membrane distillation (MD) has recently gained increasing attention because of its theoretical ability completely reject non-volatile solutes, even when treating highly concentrated feed streams such as reverse osmosis brines [1] and industrial wastewaters [2,3].

In MD, a hydrophobic porous membrane separates a hot section (feed) from a cold section. The temperature difference across the membrane generates a partial vapor pressure difference that acts as a driving force for the process and induces a pure vapor flux through the pores [4,5].

A major advantage of MD in comparison to traditional, thermally driven separation processes is the ability to generate a pure water flux without reaching the water’s boiling point: this feature makes it possible to exploit low-grade thermal sources such as industrial waste heat and solar energy [6,7]. However, feed temperatures as high as 90 °C can be useful in some cases for peculiar applications [8].

MD membranes must satisfy different key requirements in order to be profitable in distillation plants. In particular, a high porosity is recommended for generating a large evaporation surface area that can provide high distillation fluxes [4,5]. On the other hand, the pore size must be small enough to prevent the liquid feed from entering the porous structure and flooding the membrane [9]. Another characteristic improving wetting resistance is the membrane hydrophobicity. Good results can be obtained by using material with a low surface energy, such as polytetrafluoroethylene (PTFE), polypropylene (PP), and polyvinylidene fluoride (PVDF) [10], modifying the surface character of the membrane [8,11], or enhancing the surface roughness [12].

Improving these membrane features is a technological requirement that is mandatory for upscaling MD to an industrial level [11]. Different routes are currently studied, and a possible approach is the development of mixed matrix membranes. The inclusion of particles in the polymeric material can have a beneficial effect on properties such as mechanical and thermal resistance, hydrophobicity, and fouling containment [13].

As is well known, different techniques are available to prepare porous membranes, such as non-solvent or thermally induced phase separation (NIPS or TIPS), extrusion/stretching, sintering, and electrospinning, and method selection depends on the material processed [14]. In the NIPS process, a homogeneous polymer solution is first prepared and then cast on a flat surface to create a dope layer with a uniform thickness. The film is then immersed in a non-solvent bath where solvent/non-solvent exchange takes place. The initial solution is destabilized by the solvent outflow and spontaneously separates into a polymer-rich and a polymer-poor phase [15,16,17].

Among all the possible hydrophobic polymers, PVDF is one of the most studied and used to prepare MD membranes. It is characterized by outstanding chemical and thermal stability as well as good hydrophobicity. Moreover, PVDF can be easily dissolved in many common organic solvents, and membrane manufacturing can be simply carried out by NIPS or TIPS techniques [11,18].

However, the use of organic solvents raises concerns regarding the environmental sustainability and safety of the membrane preparation process. In fact, the most widely used solvents for PVDF dissolution, such as *N*,*N* dimethylacetamide (DMAc) [19], *N*,*N* dimethylformamide (DMF) [20], and *N* methylpyrrolidone (NMP) [21], have been listed as substances of very high concern (SVHC) by the European Chemical Agency (ECHA) [22]. In particular, NMP has been found to be toxic to reproduce, and, in 2018, it was included in Annex XVII of REACH (Registration, Evaluation, Authorisation and Restriction of Chemicals). Starting in 2020, the trade of NMP in the European Union has been subjected to severe limitations, and its use has been restricted [23]. In this context, green chemistry principles suggest the use of solvents with the lowest environmental impact throughout their entire life cycle (LC), from production to disposal [24]. Therefore, replacing traditional solvents with green substitutes has become a major topic in membrane preparation research [25], and many studies on different alternative solvents have been carried out in the last few years [25,26,27,28,29]. Russo et al. [27] used a commercial green solvent, namely Tamisolve^®^ NxG (Taminco, Gent, Belgium), to prepare PVDF membranes and investigated the role of the concentration of the polymer as well as of two pore-forming agents. Modifying the preparation conditions, membranes with different structures were obtained. Low PVDF concentrations led to the formation of macrovoids, and membranes with a high porosity and a large pore size were obtained, while higher polymer amounts generated spherulitic and symmetric structures. Marino et al. [29] prepared PVDF membranes using TEP for MD applications. In their work, pore forming agents greatly affected the membrane structure and performance.

Another strategy aimed at upgrading the environmental sustainability of the membrane at the use stage during its LC is to use solar energy to provide the needed heat to the feed [30,31,32]. A particular approach is the so-called direct solar membrane distillation, in which the liquid is heated directly inside the distillation cell. With this configuration, temperature polarization effects along the membrane module are almost eliminated, and the efficiency of the process is enhanced [33]. A membrane suitable for such an application must be able to directly convert the solar energy into usable heat concentrated on the membrane surface. To this end, several researchers have tested membranes coated with films containing different kinds of nanoparticles, such as silica-gold nanospheres [33] and carbon black [34], to directly heat the feed solution.

In this work, we tried to address both of the environmental issues outlined above, enhancing the safety at the preparation stage and the energy efficiency during membrane operation. Highly porous PVDF membranes were prepared using a green solvent such as triethyl phosphate (TEP), while carbon black (CB) was selected as a filler for the polymeric matrix because of its low cost, its great absorbance over the entire solar spectrum, and its non-toxicity. These characteristics made CB a perfect candidate to be integrated into the PVDF-based structure to improve the photothermal performance of the membrane. To the best of our knowledge, only one study has directly included CB particles in a PVDF membrane matrix for distillation purposes [35]. In our study, we investigated a completely different solvent/non-solvent system. A preliminary assessment of the photothermal performance of the prepared membranes was also carried out.

## 2. Material and Methods

### 2.1. Dope Solution Preparation

The dope solutions for PVDF-based membranes were obtained dissolving a certain amount of PVDF (Solef^®^ 6010, Solvay Speciality Polymers, Bollate, Italy, M_w_ 300 kDa) in triethyl phosphate (TEP, Merck, Darmstadt, Germany).

For CB-loaded membranes, the procedure was slightly different, since a uniform CB/TEP dispersion had to be prepared before adding the polymer. First, a precise amount of CB (Vulcan^®^ XC72R, Cabot Corp, Boston, MA, USA, primary particle size: 30–60 nm [36]) was weighed on an analytical balance inside a 25 mL bottle, and 15 g of TEP were added. To improve the dispersion of the CB particles, the bottles were immersed for 30 min in an ultrasonic bath. The PVDF was weighed in a different bottle, and the dispersion was added to the solid PVDF. The CB dispersion was transferred quantitatively to the ternary system, so obtained using TEP until the desired PVDF concentration was reached. The dissolution of PVDF in the TEP/CB dispersion was carried out on a stirring plate heated at 70 °C for 8 h.

### 2.2. Membrane Preparation

The membranes were prepared following the protocol described in previous works [37,38]. 

First, a commercial non-woven support (PET Viledon^®^ FO-2401, Freudenberg, Weinheim, Germany) was attached on a flat glass with adhesive tape and then impregnated with the solvent to improve the solution penetration, increasing the adhesion between the two materials. A 300 μm dope film was cast using a doctor blade. The glass plate was then immersed into an ethanol 96 *v*/*v*% non-solvent bath at a constant speed and left to precipitate for 2 h.

Finally, the solidified membrane was separated from the glass plate, rinsed with water to remove ethanol, and subsequently dried at room temperature overnight. Two different polymer concentrations and six filler amounts were explored. Table 1 summarizes the preparation conditions of all the membranes assessed in this work.

### 2.3. Membrane Characterization

The extent of dispersion of CB in the polymeric matrix was evaluated using an optical microscope (AM4515T5 EDGE, Dino-lite, Almere, The Netherlands), while the morphology of the cross section and of the membrane surface was investigated with field emission scanning electron microscopy (FE-SEM Zeiss SUPRA 40 VP, Carl Zeiss, Oberkochen, Germany). For the surface analysis, the samples were simply attached to the stub with an electrically conductive adhesive tape and then covered with a thin carbon layer by means of a high vacuum evaporator (Polaron 6700, USA). In order to study cross sections, a fragile fracture of the sample was produced in liquid nitrogen. The samples were then mounted vertically and coated with the same procedure outlined above. The images were obtained using both a conventional and an In-lens detector for secondary electrons. The acceleration voltage was set at 5 kV to prevent beam-induced modifications of the sample.

Membrane hydrophobicity was assessed using a digital tensiometer (Attension Theta, Biolin Scientific, Gothenburg, Sweden). The instrument automatically generated a 3 μL water drop that was then deposited on the surface of the membrane. For each sample, 3 drops in different spots were analyzed, and 150 contact angle values were collected for each drop over a 10 s time interval.

The mean pore size and its distribution were measured using a liquid–liquid displacement porometer (LLDP) built in the laboratory and previously described elsewhere [39]. After preliminary investigations, water and 1-octanol were selected as the displacing and wetting liquid, respectively. Their interfacial tension at 20 °C is 8.5 mN/m [40]. The membranes were first immersed in the organic phase under a vacuum to promote the flooding of the porous structure, and then mounted inside an adequate test cell. A water flux was then forced through the membrane using a HPLC syringe pump (ISCO 260D, Teledyne ISCO, Lincoln, NE, USA), and the equilibrium pressure was measured. The water flux was automatically increased stepwise, and an equilibrium pressure for each flux was measured and correlated to the pore size using the Laplace equation:

(1) $$r=\frac{2{B\gamma }_{l}\cos\theta }{P}$$

where B is a geometric factor accounting for the pore shape (0 < B < 1 for non-cylindrical shapes; B = 1 for cylindrical pores), γ_l_ is the liquid–liquid interfacial tension, r is the pore size, and θ is the contact angle between the membrane and the wetting liquid feed.

The determination of the total membrane porosity (ε%) was performed with a gravimetric method. A small piece of dry membrane was detached from the support material and weighed on an analytical balance. The samples were then impregnated using 1-octanol under a vacuum and weighed again. The total porosity was then calculated using the following equation:

(2) $$\varepsilon \%=\frac{V_{\mathrm{empty}}}{V_{\mathrm{tot}}}\cdot 100=\frac{\frac{\left(m_{w}-m_{d}\right)}{\rho_{\mathrm{oc}}}}{\frac{m_{d}}{\rho_{\mathrm{pol}}}+\frac{\left(m_{w}-m_{d}\right)}{\rho_{\mathrm{oc}}}}\cdot 100$$

where m_w_ and m_d_ are the masses of impregnated and dry membrane, respectively; ρ_oc_ and ρ_pol_ are the 1-octanol density (0.83 g/cm^3^) and the PVDF density (1.8 g/cm^3^) at 25 °C.

The infrared spectroscopy analyses were performed using a Vertex 70 (Bruker, Billerica, MA, USA) FT-IR spectrometer operated in ATR (attenuated total reflection) mode.

To measure the liquid entry pressure (LEP), the membrane was put in a sample holder (diameter: 24 mm) containing deionized water. The cell pressure was increased stepwise at 5 min intervals using compressed air and was measured using a digital manometer (Digitron 2026P, Ferentino, Italy). The LEP value was registered when the first waterdrop passed through the membrane.

**Figure 1:**
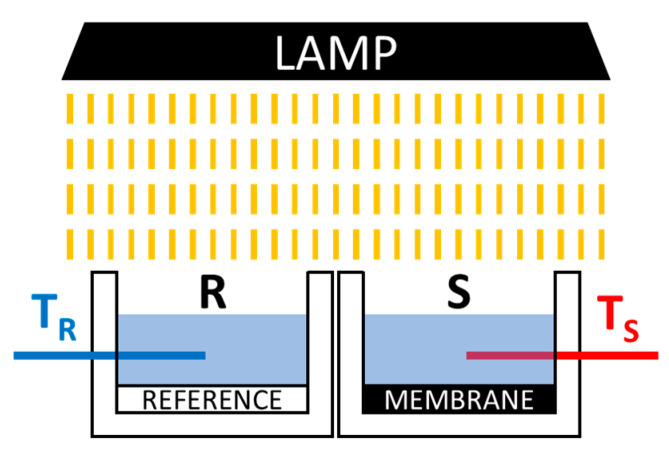
Sketch of the setup for membrane photothermal tests. T_R_: reference thermocouple; T_S_: sample thermocouple.

The evaluation of the photothermal properties of the membranes was performed using a simple equipment built for the purpose, schematized in Figure 1.

The setup was composed of two geometrically equal containers (S and R), equipped with a thermocouple (T_S_ and T_R_) and built using an expanded PVC in order to assure good thermal insulation from the environment. A light source with a solar-like emission spectrum (Milyn 100 W LED lamp, radiant power 11.3 W) was placed 19 cm above the sample and reference containers. The tested sample (area: 17.5 cm^2^) was glued with double-sided tape inside the S container, while a pure PVDF membrane was placed similarly in the R container. Twenty grams of deionized water at room temperature were then poured inside both tanks. To start the measurements, the light was turned on, and the temperature data acquisition was started and set to collect both T_S_ and T_R_ for at least 30 min at 15 s intervals.

The heat per unit area generated by the absorption of light for the membrane and the reference was calculated using the following equation [41]:

(3) $$\frac{Q}{A}=\frac{c\cdot m\cdot \left(T-T_{0}\right)}{A}$$

where c is the specific heat of water, m is the water mass, T is the water temperature at a given time, T_0_ is the starting water temperature, and A is the membrane surface area.

The heat correlated to the presence of the carbon black filler (Q_CB_) was obtained as the difference between the heat obtained in the sample chamber (Q_S_) and that produced in the reference (Q_R_). Since the starting temperature and the exposed membrane surfaces were the same, the heat was directly proportional to the difference of temperature between the sample and the reference chamber, as expressed by the following equation:

(4) $$\frac{Q_{\mathrm{CB}}}{A}=\frac{Q_{S}-Q_{R}}{A}=\frac{c\cdot m\cdot \left(T_{S}-T_{R}\right)}{A}$$

### 2.4. Membrane Performance Evaluation

**Figure 2:**
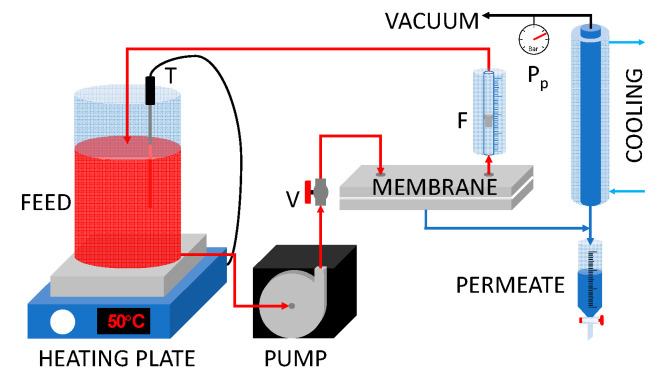
VMD setup scheme. V: valve for the flow regulation; F: flow meter; P: vacuum meter; T: temperature control probe.

The distillation performance of the membranes was evaluated using a vacuum membrane distillation (VMD) setup, schematized in Figure 2.

Two liters of feed (deionized water or a 90 g/L NaCl solution) were poured into a glass reservoir and heated using a thermostatic heating plate. The feed was sent to the membrane module using a centrifugal pump, and the retentate was returned to the reservoir. The recirculation flowrate (200 L/h) was controlled by means of a spherical valve and measured with a flowmeter mounted on the membrane cell outlet.

VMD configuration requires the application of a low pressure on the permeate side of the membrane in order to increase the driving force of the process [42]. To this end, the permeate side of the membrane cell was kept at an absolute pressure of 25 mbar by a vacuum pump. The vapor extracted from the membrane cell flowed through a series of glass condensers (cooled using a water/glycol solution at 0.1 °C) and was collected as liquid water in a graduated dropping funnel. The permeate flux was calculated on the basis of the volume of water collected during fixed time intervals. The separation performance of the membrane was evaluated measuring the electrical conductivity of both the liquid feed and the distillate, and the salt rejection (R%) was calculated as

(5) $$R\%=\frac{\sigma_{f}-\sigma_{d}}{\sigma_{f}}\cdot 100$$

where σ_f_ and σ_d_ are the electrical conductivities of the feed and the distillate, respectively. Table 2 summarizes the operating conditions used for all the VMD tests.

## 3. Results and Discussion

### 3.1. Effect of Solvent Selection and of PVDF Concentration

**Figure 3:**
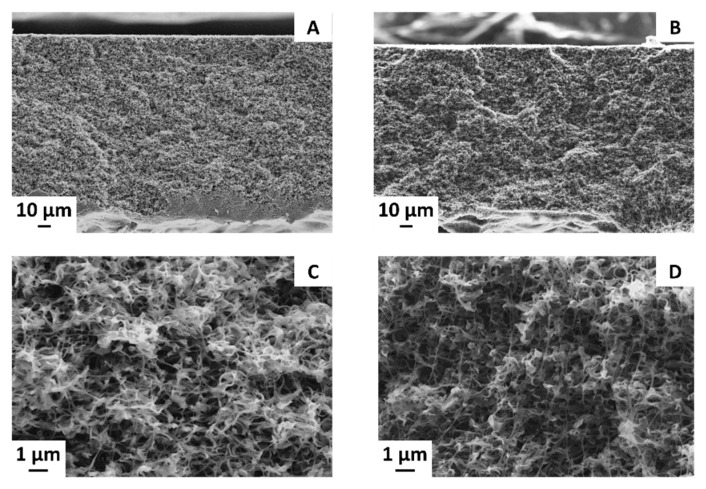
Cross-section images of (**A**,**C**) 145_0 and (**B**,**D**) 16_0 membranes.

Polymer concentration is one of the most important factors that influence the membrane structure [17]. Figure 3 reports the cross section of the membranes prepared with 14.5 wt % (A and C) and 16 wt % (B and D) PVDF in TEP, without the addition of CB.

An increase of the PVDF concentration in the dope solution had minor effects on the morphology of the final membranes. The main changes consisted in a decrease of the mean pore size and of the porosity of the membrane, as it will be discussed further in the following section. The 145_0 and 16_0 membranes consisted in a honeycomb structure characterized by small, tortuous, and uniform interconnected cavities. Crystal elements formed by interlocked lamellae and fibrils constitute the polymeric matrix. An almost identical structure was reported by other researchers who also prepared PVDF/TEP membranes [29,43,44]. It was, however, observed that, in a harsh nonsolvent such as water, a dense skin is formed that hinders the water vapor flux, whereas, in a soft nonsolvent, such as the ethanol used in this work, a very porous top surface is obtained, which enhances the water transport.

In previous research carried out with DMF by our group [37], a similar morphology was obtained. With ethanol as the main constituent of the nonsolvent bath, a totally symmetric membrane was created without a skin layer. The polymeric network was well interconnected, and some spherulitic structures with very small dimensions were detectable in this case. Therefore, provided that a weak nonsolvent is used, TEP is highly successful in replacing more hazardous solvents such as DMAc and DMF, as symmetric highly porous membranes with a uniform bi-continuous morphology are easily obtainable with this greener substitute.

PVDF is a semi-crystalline polymer that exhibits polymorphism [45]. It can give rise to five different crystalline forms, among which the α and β phases are the most common. The chains of the apolar α-phase have a TGTG conformation, while the polar β phase has a parallel arrangement of all-trans chains. Recent literature shows that differences in the polymorphic phase could have an effect on the final membrane properties, so this aspect was investigated. Some authors [46] claim that PVDF membranes dominated by the α phase, prepared using TEP as the solvent, exhibit a very high permeability. Other authors [47] have reported that the piezoelectric properties associated with the PVDF β phase significantly reduce membrane fouling.

During the crystallization process starting from a homogeneous solution, the predominance of one phase over the others is induced by the preparation conditions and by the affinity between the polymer segments and the solvent molecules [46]. The solvent role can be predicted taking into account its solubility parameters. Table 3 reports the Hansen solubility parameters for PVDF and two solvents: DMF and TEP.

The α form of PVDF is kinetically favored, and it will be preferentially formed in the separation phase process, which is strongly affected by kinetic factors. Nevertheless, the crystallization of the β form can be promoted using a polar solvent [49,50,51]. This is due to the formation of molecular interactions capable of stabilizing the net dipole arising from the TTTT chain conformation in the β phase. In particular, hydrogen bonds are the strongest interactions. In Table 3, δ_h_ (DMF) is noticeably greater than δ_h_ (TEP), and this is also true for δ_p_. Against this background, TEP should promote the α phase formation, and DMF should enhance the β phase formation. FT-IR analyses were used to investigate the possible different effects of the solvent on the PVDF polymorphism.

**Figure 4:**
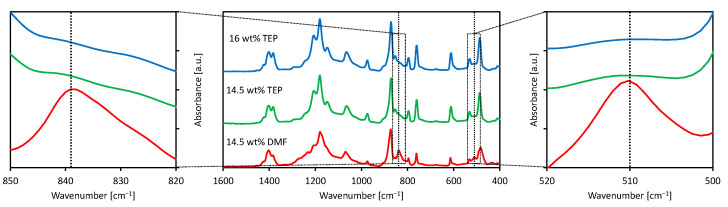
FT-IR spectra of the 16 wt % PVDF (blue) and 14.5 wt % PVDF (green) samples (this work) compared with a 14.5 wt % PVDF membrane prepared using DMF as solvent (red).

Figure 4 reports the FT-IR spectra of the 145_0 and 16_0 membranes prepared using TEP, in comparison with the spectrum of a sample prepared in identical conditions during a previous work, but using DMF as a solvent [37].

The spectra of the 145_0 and 16_0 samples, obtained from PVDF/TEP dope solutions, were characterized by highly intense signals that are typical of the α phase (531, 610, 795, and 975 cm^−1^ [45]). For both membranes, no peaks correlated with the β phase were found. In the spectrum of the sample prepared with DMF as a solvent, the signals of both α and β phases were clearly discernible. In particular, there were new signals that are characteristic of the β phase at 511 and 840 cm^−1^ (see the enlargements in Figure 4) and at 1431 cm^−1^. This finding indicates the orienting action performed by DMF on the polymeric chains, which led to the coexistence of both phases in the crystallized matrix.

### 3.2. CB Distribution in the PVDF Membranes

**Figure 5:**
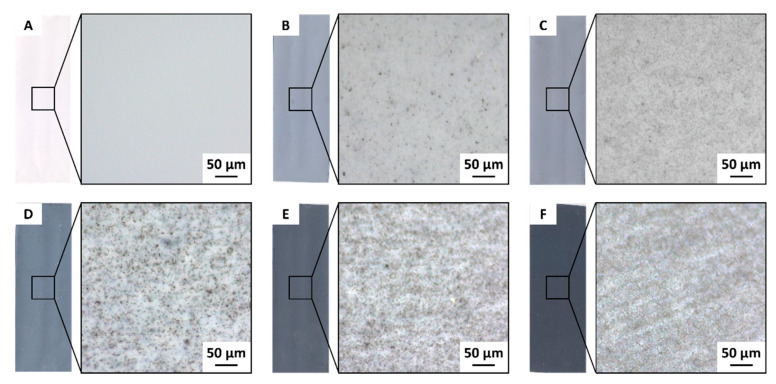
Real size (left) and optical microscopy images (right) of the surface of the 14.5 wt % PVDF membranes with (**A**) 0 wt % CB, (**B**) 0.5 wt % CB, (**C**) 1 wt % CB, (**D**) 2 wt % CB, (**E**) 5 wt % CB, and (**F**) 7.5 wt % CB.

One of the key parameters in mixed matrix membranes is the distribution of the filler particles in the polymer matrix. A simple method to roughly evaluate the uniformity of the filler dispersion and the eventual formation of aggregates when using carbon-based particles is optical microscopy, since the black color of the filler can be easily detected on the white background exhibited by the porous polymeric matrix. Figure 5 shows the images of the membranes prepared with 14.5 wt % PVDF and different CB loadings.

The increase of the CB loading had an intense effect on the grey intensity of the membranes, shown in Figure 5. The pure PVDF membrane was completely white, while the addition of CB induced first a light grey color that became darker as the filler concentration increased (see the left photograph for each sample). The 145_75 sample was almost black. The microscopy investigations on the membranes prepared with the lower amounts of CB (Figure 5B,C) showed a progressive increase in the concentration of CB. The dispersion of the filler remained uniform even for the films prepared with a larger filler loading (Figure 5D–F). Only small clusters were present, and they were evenly spread over the entire membrane surface. It is known that native CB nanoparticles are typically fused into chain-like aggregates. Higher magnifications confirmed that the sonication treatment performed on the CB/TEP mixture was enough to well disperse the filler inside the solvent. Moreover, using the filler dispersion to dissolve the polymer allowed for the generation of membranes with an even distribution of CB.

### 3.3. Effect of CB Loading on the Membrane Structure

**Figure 6:**
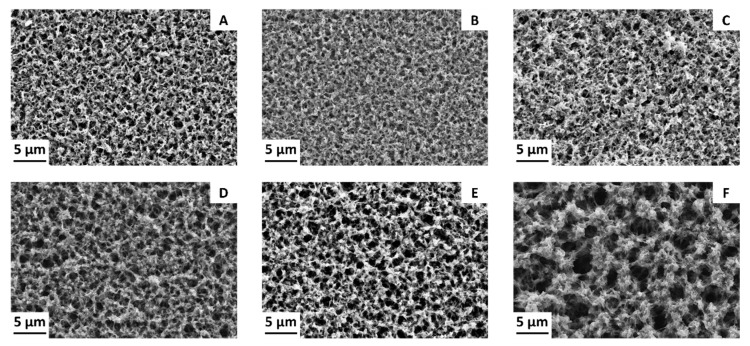
FE-SEM images of the surface of the 14.5 wt % PVDF membranes with (**A**) 0 wt % CB, (**B**) 0.5 wt % CB, (**C**) 1 wt % CB, (**D**) 2 wt % CB, (**E**) 5 wt % CB, and (**F**) 7.5 wt % CB.

The addition of CB also had a great influence on the membrane surface morphology, as shown in Figure 6, which shows FE-SEM images of the 14.5 wt % PVDF samples.

The introduction of small amounts of CB reduced the surface pore size of the membranes (Figure 6B,C), while further increases of the filler loading led to increases in the pore size. Measured mean pore sizes are summarized in Table 4. These differences were confirmed by other analyses carried out on the same samples. A summary of the main characteristics of these membranes is reported in Table 4.

The pore size determination, carried out with the LLDP technique, and the total porosity measurements confirmed the trend seen during the FE-SEM observations and LEP measurements. Small amounts of CB had an adverse effect on both pore size and porosity, while larger filler concentrations generated more open structures. Since these are two important factors influencing mass transfer through the membranes, it is expected that a larger pore size and porosity values translate into higher transmembrane vapor fluxes during MD operations.

Nevertheless, an increase in pore size could facilitate the intrusion of the liquid feed inside the membrane, leading to a decrease in both the distillate flux and the separation ability of the process. One parameter that counteracts this tendency is the membrane’s hydrophobicity. A small but significant improvement of the contact angle was registered as the CB loading increased, particularly for the 14.5 wt % PVDF samples, as shown in Table 4.

### 3.4. Photothermal Properties

**Figure 7:**
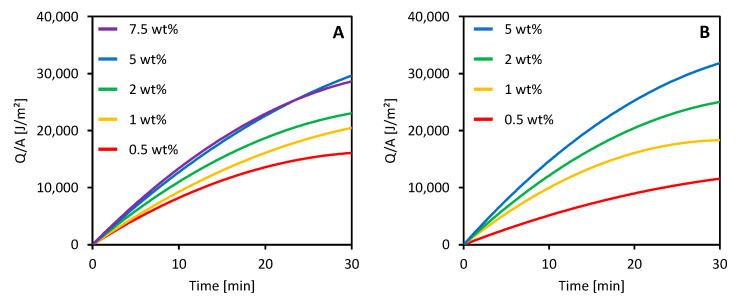
Heat generated by the irradiated membranes prepared with (**A**) 14.5 wt % and (**B**) 16 wt % PVDF and different CB loadings.

Some preliminary tests on the photothermal properties of the CB-loaded membranes were carried out using the setup schematized in Figure 1. The heat generated per unit area (Q/A) and transferred to the water in the container is illustrated in Figure 7 for 14.5 wt % and 16 wt % PVDF membranes with different CB loadings.

All the membranes prepared by adding CB to the dope solution were able to absorb the irradiated light and to convert it into heat that was then transferred to the water contained in the sample holder. The heat produced by the membranes increased over time and showed a tendency to reach an equilibrium value. The behavior of the membranes was already well defined after 30 min of light irradiation. An increase in CB loading improved the photothermal performance of the membranes for both 14.5 wt % and 16 wt % membranes. However, CB concentrations higher than 5 wt % did not cause any improvement. A similar behavior was previously observed by other researchers who used CB to create a photoactive layer over commercial PVDF membranes [52].

These preliminary experiments suggested a possible use of simply prepared CB-loaded PVDF membranes in direct solar MD application. A deeper and more complete research activity is currently being developed in our laboratories, and the results will be described in upcoming publications.

### 3.5. MD Performance

**Figure 8:**
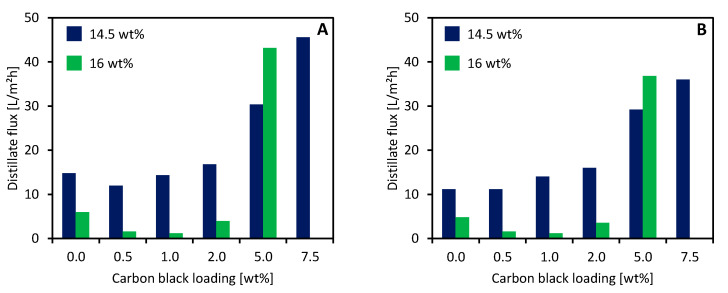
Mean distillate fluxes obtained with CB-loaded membranes using (**A**) pure H_2_O and (**B**) a 90 g/L NaCl solution as feed.

All the prepared membranes were tested in the VMD setup outlined in Section 2.4. Tests with pure water and a concentrated NaCl solution yielded measurements of both the distillate flux and the separation ability. The VMD mean distillate fluxes are illustrated in Figure 8.

The addition of CB to the dope solution greatly influenced the performance of the membranes during the VMD operation. At low concentrations, CB addition caused a decrease in the distillate flux for both 16 wt % and 14.5 wt % PVDF membranes. However, further increases in CB induced a tremendous increase in the VMD performance.

These data are in accordance with the porosity values and the mean pore sizes reported in the previous section (Table 4). However, our results are apparently in disagreement with some literature data about mixed matrix membranes. Zhao et al. [53] added activated carbon (AC) to A PVDF/DMAc dope solution used for the preparation of hollow fiber membranes. In their work, AC increased the membrane porosity and the VMD performance at low concentrations (<0.09 wt %); when higher amounts were added to the dope solution, an inverted trend was registered (i.e., porosity and VMD flux decreased).

The difference can be related to the filler concentration range that was explored in their work and ours. In fact, regarding the CB concentration of the whole solution mass, CB amounts between 0.07 wt % and 1.09 wt % were assessed in this paper, while Zhao et al.’s tests were carried out between 0.03 wt % and 0.15 wt % AC.

The distillate flux obtained with 145_0 was one order of magnitude higher in comparison with that of a commercial PVDF membrane, as reported in Table 5.

The great performance difference could be influenced by the vacuum pressure applied. In fact, lower values increase the driving force of the process and the distillate flux. However, it is difficult to find literature data obtained under the same operating conditions. Nevertheless, these results confirm that using TEP as a green solvent is a viable solution to develop new membranes aimed at MD applications.

The experiments carried out with a 90 g/L NaCl solution confirmed the trend registered with pure water for the distillate flux. Moreover, they allowed the salt rejection (i.e., the separation ability) of the membranes to be calculated using Equation (5). For all tests, the electrical conductivity of the distillate never exceeded 5 μS/cm, meaning that a full salt rejection was obtained by all the membranes.

## 4. Conclusions

This work aimed at studying some possible routes to improvements in the sustainability of the MD technique. TEP was selected to prepare PVDF membranes, replacing the traditional solvents and reducing the impact on human health and the environment. Samples with a high porosity and a high hydrophobicity were obtained.

Including CB inside the dope solution allowed for modifications of some of the main properties of the membranes. At low concentrations (<2 wt %), CB worsened the membrane’s performance, due to a reduction in both the pore size and porosity of the samples.

Larger amounts of CB had the opposite effect. When the filler concentration was raised to 5 wt % or more, marked increases in the pore size and porosity of the membrane were registered. These structural changes improved the distillation performance, namely, the distillation flux, without affecting the separation ability of the process.

Finally, some preliminary data on the photothermal properties of the membranes were collected. The samples containing CB were able to absorb light and convert it into heat. This feature laid the foundations for the use of such membranes in solar enhanced distillation configurations that, by exploiting an inexhaustible energy source, can considerably improve the sustainability of the process.

## Figures and Tables

**Table 1:** Preparation conditions of the tested membranes.

Sample	16_0	16_05	16_1	16_2	16_5	145_0	145_05	145_1	145_2	145_5	145_75
CB concentration ^1^ [wt %]	0	0.5	1.0	2.0	5.0	0	0.5	1.0	2.0	5.0	7.5
PVDF concentration [wt %]	16					14.5					
Solvent		TEP									
Non-solvent		EtOH 96 *v*/*v*%									
Casting temperature [°C]		25									
Casting thickness [μm]		300									

^1^ with respect to PVDF mass.

**Table 2:** VMD tests conditions.

Tested Feeds	Deionized Water NaCl Solution 90 g/L
Feed temperature	50 °C
Feed flowrate	200 L/h
Membrane area	25 cm^2^
Vacuum pressure	25 mbar

**Table 3:** Hansen solubility parameters of PVDF, DMF, and TEP.

	δ_d_ [MPa^½^]	δ_p_ [MPa^½^]	δ_h_ [MPa^½^]	δ_T_ [MPa^½^]
PVDF [48]	17.2	12.5	9.2	23.2
DMF [48]	17.4	13.7	11.3	24.8
TEP [48]	16.8	11.5	9.2	22.3

**Table 4:** Main properties of 14.5 wt % and 16 wt % PVDF membranes.

Sample	Pore Size FE-SEM [μm]	Pore Size LLDP [nm]	LEP [μm]	Porosity [%]	Contact Angle [°]
145_0	0.9 ± 0.4	99 ± 7	6.3 ± 0.2	76 ± 3	128 ± 3
145_05	0.7 ± 0.2	86 ± 16	5.8 ± 0.1	68 ± 4	139 ± 4
145_1	0.8 ± 0.3	167 ± 8	5.3 ± 0.3	77± 1	127 ± 5
145_2	1.1 ± 0.4	191 ± 11	5.5 ± 0.1	80 ± 1	143 ± 1
145_5	1.4 ± 0.2	282 ± 19	4.8 ± 0.1	85 ± 1	143 ± 1
145_75	2.1 ± 0.7	857 ± 21	4.1 ± 0.3	87 ± 1	145 ±6
16_0	0.9 ± 0.2	54 ± 1	6.2 ± 0.3	73 ± 2	132 ± 4
16_05	0.6 ± 0.1	29 ± 1	6.3 ± 0.3	70 ± 2	126 ± 5
16_1	0.5 ± 0.1	43 ± 3	6.0 ± 0.1	69 ± 1	134 ± 5
16_2	0.7 ± 0.2	98 ± 3	6.2 ± 0.2	78 ± 1	129 ± 3
16_5	1.5 ± 0.5	498 ± 58	4.8 ± 0.2	85 ± 1	139 ± 3

**Table 5:** Comparison of VMD performance between a commercial PVDF membrane and the 145_0 sample under similar operation conditions.

Membrane	Feed Temperature	Vacuum Pressure	Distillate Flux	Reference
145_0	50 °C	25 mbar	11.2 L/m^2^h	This work
Millipore 0.22 µm	50 °C	100 mbar	1.3 kg/m^2^h	[54]

## Data Availability

Not applicable.
